# Internet-based treatment for vulvodynia (EMBLA) – Study protocol for a randomised controlled study

**DOI:** 10.1016/j.invent.2021.100396

**Published:** 2021-04-20

**Authors:** Andrea Hess Hess Engström, Merit Kullinger, Izabella Jawad, Susanne Hesselman, Monica Buhrman, Ulf Högberg, Alkistis Skalkidou

**Affiliations:** a Centre for Clinical Research, Västmanland County Hospital, Västerås, Sweden; b Department of Women's and Children's Health, Uppsala University, Uppsala, Sweden; c Center for clinical research, Uppsala University, Falun, Sweden; d Department of Psychology, Division of Clinical Psychology, Uppsala University, Uppsala, Sweden

**Keywords:** ACT, Acceptance and commitment therapy, CBT, Cognitive Behavioural therapy, CPAQ, Chronic Pain Acceptance questionnaire, FSFI, Female Sexual Function Index, FSDS-R, Female Sexual Distress Scale – Revised, BAI, Beck Anxiety Inventory, MADRS-S, Montgomery-Åsberg Depression Rating Scale, rDAS, Revised Dyadic Adjustment Scale, SWLS, Satisfaction with Life Scale, EQ5-D, EuroQol-5-dimension questionnaire, ESSI, ENRICHD Social Support Instrument, LOCF, last observation carried forward, Vulvodynia, Acceptance and commitment therapy, Internet-based, Randomised controlled trial

## Abstract

**Background:**

Vulvodynia is defined as vulvar pain for at least 3 months without a clear cause. To the best of our knowledge, there are no trials investigating the effects of internet treatment using CBT (Cognitive behavioural therapy) treatment with Acceptance and Commitment Therapy (ACT) components for women with vulvodynia. The aim of this study is to examine the effects of such a guided internet-based intervention on provoked vulvar pain during the waiting period before clinical treatment.

**Methods:**

We will randomise 52 patients to either guided internet-based intervention with CBT with (ACT) components or no intervention during the waiting period for treatment as usual. Online assessments are conducted at baseline, posttreatment, and at follow-up after 9 months. The primary outcome measure is provoked vulvar pain. Secondary outcomes are depression, anxiety, sexual function, and quality of life. Linear-mixed effect models will be used to assess the effect of the internet-based intervention on vulvar pain, pain acceptance, depression, anxiety, sexual function, and quality of life over time, by applying the intention-to-treat approach. Continuous data will be analysed with general linear models using intention-to-treat and also per protocol approaches to assess the effects of the intervention at different time points. Ordinal and binary data will be analysed with Mann Whitney's test, Fischer's exact test and multivariate logistic regression, respectively.

**Discussion:**

As a randomised controlled trial with short- and long-term follow-up points, the EMBLA study intends to provide a novel and better understanding regarding the treatment of vulvodynia and the role of internet-based treatment as a complement to standard care for women suffering from vulvodynia. The effects of vulvodynia on pain, sexual function, quality of life, depression, and anxiety are investigated. The study's results are expected to be of value in the planning of clinical care in the medical area. High dropout rates and technical difficulties associated with using the platform are common in similar studies.

**Trial registration number:**

NCT02809612

## Highlights

• A randomised trial can give a better understanding of treatment for vulvodynia.
• The effects of internet-based treatment will be investigated.
• Well-accepted self-assessment questionnaires are used.
• High dropout rates and technical difficulties are common in similar studies.

## 1 Introduction

Vulvodynia is defined as vulvar pain lasting for at least 3 months without a clear cause (Bornstein et al., 2016). In provoked vulvodynia, pain is experienced upon mechanical stimuli such as the use of a tampon or vaginal intercourse (Bohm-Starke, 2010; Bornstein et al., 2016; Bornstein et al., 2019; Henzell and Berzins, 2015). The prevalence of vulvodynia varies between studies, but it has been estimated at 10–28%, affecting mostly women between 20 and 30 years (Bohm-Starke, 2010; Reed et al., 2012; Vieira-Baptista et al., 2014; Pukall et al., 2016; Reed et al., 2012; Sadownik, 2014). The aetiology is not clear, but a combination of psychosocial and pathophysiological mechanisms is believed to contribute to the onset (Bergeron et al., 2020; Bornstein et al., 2016; Faye and Piraccini, 2020). Regarding treatment, a multidisciplinary approach is recommended, and pelvic floor physiotherapy and cognitive behavioural therapy (CBT) are considered first line treatment (Bergeron et al., 2020; De Andres et al., 2016; Rosen and Dawson, 2019; Sadownik, 2014, van der Meijden et al., 2017).

Some trials have investigated the effects of CBT for patients with vulvodynia and found positive results on pain, sexual function, psychological adjustment, and treatment satisfaction for patients with vulvodynia (Bergeron et al., 2001; Bergeron et al., 2016; Goldfinger et al., 2016; Lindstrom and Kvist, 2015; Masheb et al., 2009). There are also promising results regarding CBT intervention delivered via the internet for women with sexual dysfunction, including genital pain (Jones and McCabe, 2011). Further, in two trials regarding mindfulness cognitive therapy, also called third wave cognitive behavioural therapy, an effect, similar to CBT, on pain and sexual function is reported (Brotto et al., 2015; Guillet et al., 2019).

Whilst some research has been made on the effects of CBT for vulvodynia, knowledge about the effects of Acceptance and Commitment Therapy (ACT), also part of third wave cognitive behavioural therapy, on provoked vulvodynia is lacking. ACT offers an alternative to traditional attempts to control unwanted psychological experiences by developing acceptances skills to regulate behaviour (Zhang et al., 2017). Instead of trying to control form and frequency of thoughts and feelings, the focus of ACT is on improving functioning and decreasing interference of pain by altering the function, i.e. by increasing psychological flexibility, which can be defined as the ability to act in accordance with one's values and goals despite pain (Feliu-Soler et al., 2018; Hayes and Lillis, 2013; Hayes et al., 1999; Hayes et al., 2006; McCracken and Vowles, 2014; Trompetter et al., 2015a, Trompetter et al., 2015b; Vowles and McCracken, 2008; Vowles et al., 2014). There are six core processes in ACT: 1) acceptance (embrace private events without attempts to change their frequency or form), 2) cognitive defusion (attempt to alter undesirable functions of thoughts without trying to alter their frequency or form), 3) being present (non-judgmental contact with events as they occur), 4) self as context (awareness of one's own experiences without attachment to them), 5) values (choose life directions), and 6) committed action (act according to one's values) (Hayes et al., 2006).

Previous research indicates that ACT is more effective than no treatment for chronic pain (Hann and McCracken, 2014; Hughes et al., 2017; Yu and McCracken, 2016). There are also promising results for ACT delivered via the internet (Buhrman et al., 2013; Buhrman et al., 2015; Carlbring et al., 2018; Lin et al., 2017; Trompetter et al., 2015a, Trompetter et al., 2015b), but knowledge about if it is suitable for vulvar pain is lacking. Cognitive behavioural factors are associated with experience of pain and sexual functioning, suggesting that addressing emotional and sexual cognitive factors is important in vulvodynia management (Chisari and Chilcot, 2017; Chisari et al., 2021).

Vulvodynia has a negative impact in women's well-being and psychosexual function (van der Meijden et al., 2017), and the care available may vary depending on local resources (Bergeron et al., 2020). Internet interventions improve accessibility of health care services (Andersson, 2018; Andersson et al., 2019; Feliu-Soler et al., 2018), but its effects have not yet been investigated in a population with vulvodynia. To fill the knowledge gap regarding internet interventions for vulvodynia, the EMBLA study was developed to investigate the effects of guided internet treatment using ACT-principles for women with vulvodynia during the waiting period before clinical treatment.

### 1.1 Primary objectives

The aim of this study is to examine the effects of a guided internet intervention on provoked vulvar pain, compared with no intervention during the waiting period before clinical treatment compared with the waiting period in conjunction with treatment.

### 1.2 Secondary objectives

The secondary objectives are to examine the effects of guided internet intervention on depression, anxiety, sexual function, and quality of life.

## 2 Methods

### 2.1 Study design

The EMBLA study is a multi-centre randomised trial initiated by the Department of Obstetrics and Gynaecology at Uppsala University Hospital, Uppsala, Sweden. The trial will be reported in accordance with the CONSORT guidelines (Schulz et al., 2010).

### 2.2 Eligibility criteria

Inclusion criteria include:

➢ Symptoms of vulvodynia for at least 6 months, and confirmation of the medical diagnosis at the gynaecologist's appointment after study recruitment from waiting lists for clinical treatment, or by the woman herself following study recruitment through social media.
➢ Access to a computer with internet connection.
➢ Age of ≥18 years.
➢ A Swedish personal identification number for access to the treatment platform.

Exclusion criteria include:

➢ Unclear diagnosis at the screening interview.
➢ Ongoing examination/treatment related to vulvodynia.
➢ Lack of fluency in Swedish, since the treatment is delivered in Swedish.
➢ Severe, acute, or untreated mental illness or substance abuse.

### 2.3 Study population and recruitment

Women have been assessed for eligibility since 2016 and the aim is to complete recruitment during 2020. Patients with vulvodynia are recruited from waiting lists for clinical treatment at gynaecological clinics in four counties in Sweden (Falun, Gävle, Uppsala, and Örebro), and through advertisements in social media. The women then receive detailed information about the study over the telephone by the research team. The same information in written form is sent by regular post. Participants are included only after a telephone screening interview performed by one of the researchers, and after they return a signed consent form. A structured questionnaire for diagnostics and evaluation of symptoms developed by the Swedish Working and Reference group for Vulva Diseases, (SFOG, 2013), and structured questions about psychological symptoms, are used for suitability screening in the study. The screening interview is usually scheduled after, but may also occur before, a gynaecologist's appointment. Participants recruited via social media are included in the study only if they can confirm a previous medical diagnosis of vulvodynia and had no ongoing treatment for vulvodynia when they enrol the study. After inclusion, patients fill out the baseline questionnaire and are then randomised to the intervention or control groups.

### 2.4 Randomisation

Participants provide informed consent at T0 and are thereafter informed about which group they are allocated to, either the internet treatment group or the control (no treatment) group. Randomisation and allocation are performed using simple randomisation. The randomisation list is created using an automated, web-based randomisation program (https://www.graphpad.com/quickcalcs/randomize2/).

### 2.5 Intervention

The treatment is provided via an internet-based secure platform with dual authentication that is approved for regular health care use and was developed by psychologists from the Pain Treatment Centre and the Department of Ear, Nose and Throat Diseases at Uppsala University Hospital in Sweden. The platform contains material such as videos, audio files, and self-assessment questionnaires. The platform enables the communication between participants and eCoaches, who are research assistants trained to provide written feedback on participants' assignments after module completion and to respond to their questions. Standard answers are used as templates to address questions and to provide feedback. If the eCoach has concerns about how to conduct treatment or write feedback, they discuss with a senior researcher. A national personal identification number and a password are necessary to securely access the platform. All communication concerning the treatment is handled within the platform. The participants can contact the eCoaches and receive a response within 3 working days. The eCoaches monitor treatment and notify the participants via reminder emails if they have been inactive for more than 4 days.

**Fig. 1:**
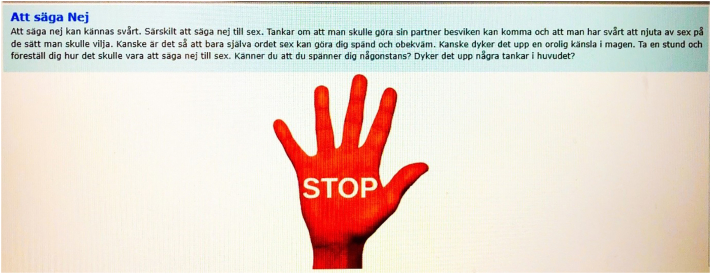
Example of information module on the option of saying no to sexual acts.

**Fig. 2:**
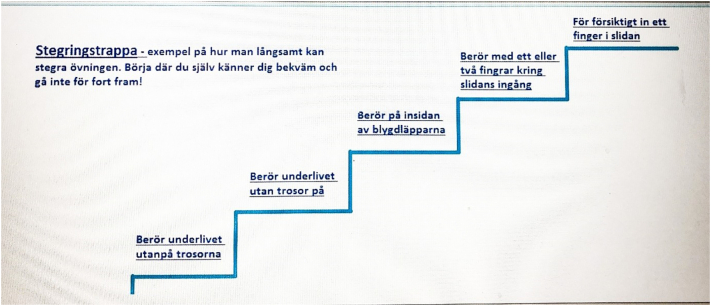
Example of exercise module on the different steps for desensitisation exercises.

The treatment consisted of six different modules, spanning a 6-week treatment period, based on an ACT manual for the treatment of patients with chronic pain (Buhrman et al., 2013), and adapted for patients with vulvodynia by a multidisciplinary team (gynaecologist, psychologist physiotherapist and midwife) with experience in treating patients with vulvodynia. The treatment was CBT-based, with ACT components, with a multidiscplinary approach; the CBT/ACT components were used as a framework during the entire treatment, for example, the modules 3 and 4 had information and exercises about valued direction and management of negative thoughts (Table 1). The modules are divided into information and treatment assignments (Figs. 1 and 2). The information modules contain knowledge about anatomy, pelvic floor muscle function, sexuality, chronic pain, and vulvodynia. Some parts of patient education are also given in video format, such as information regarding pelvic floor anatomy instruction, pelvic floor exercises is given by a physiotherapist, and information about vulvodynia, communication with partner, and sexuality is given by a midwife. The exercise modules contain exercises related to CBT-based ACT. Further, daily exercises were recommended and consisted of mindfulness, pelvic floor muscle exercises, and desensitisation exercises. The participants in the control group, on request, are given access to the internet-based treatment after 10.5 months from study inclusion. The average number of words per module is 7401 (module 1: 8143, module 2: 8760, module 3: 7638, module 4: 6755, module 5: 8813, and module 6: 4294). It is estimated that about 30 min per day are needed to complete one module per week. Written feedback, consisting of positive reinforcement related to a participant's assignment, is provided weekly to the participants on their submitted assignments after module completion by the eCoaches.

**Table 1:** Description of the six different modules included in the intervention group.

Module	Information	Exercises	Daily training
1. Introduction	Vulvodynia Chronic pain Primary and secondary suffering CBT, ACT and internet-based treatment	Primary and secondary pain Circle of pain Expectations and planning Personal commitments	Mindfulness exercise
2. Pelvic floor	Anatomy of the vulva Pelvic floor Stress and anxiety Exposure	Things I have done Willingness diary	Mindfulness exercise Pelvic floor exercise Exposure exercise
3. Values	Valued direction Willingness	What do you want your life to stand for? Life compass Why willingness? To have hope	Mindfulness exercise Pelvic floor exercise Exposure exercise
4. Thoughts	Negative thoughts Alternative attitude Sexuality	To have and to be a thought The little word”and” Reformulate goals	Mindfulness exercise Pelvic floor exercise Exposure exercise
5. Relations	Relations Sexual identity Desire Communication Exposure	Body contact Stoplight	Mindfulness exercise Pelvic floor exercise Exposure exercise
6. Maintenance	Summary of the sections The new contract Maintenance Closure	Reflections Evaluate my commitments Maintenance plan	Mindfulness exercise Pelvic floor exercise Exposure exercise

Technical support is provided by an internet support team when participants report technical problems to an eCoach. A dedicated Gmail account is added for communication with participants should technical problems with the platform arise.

### 2.6 Collection of data

**Fig. 3:**
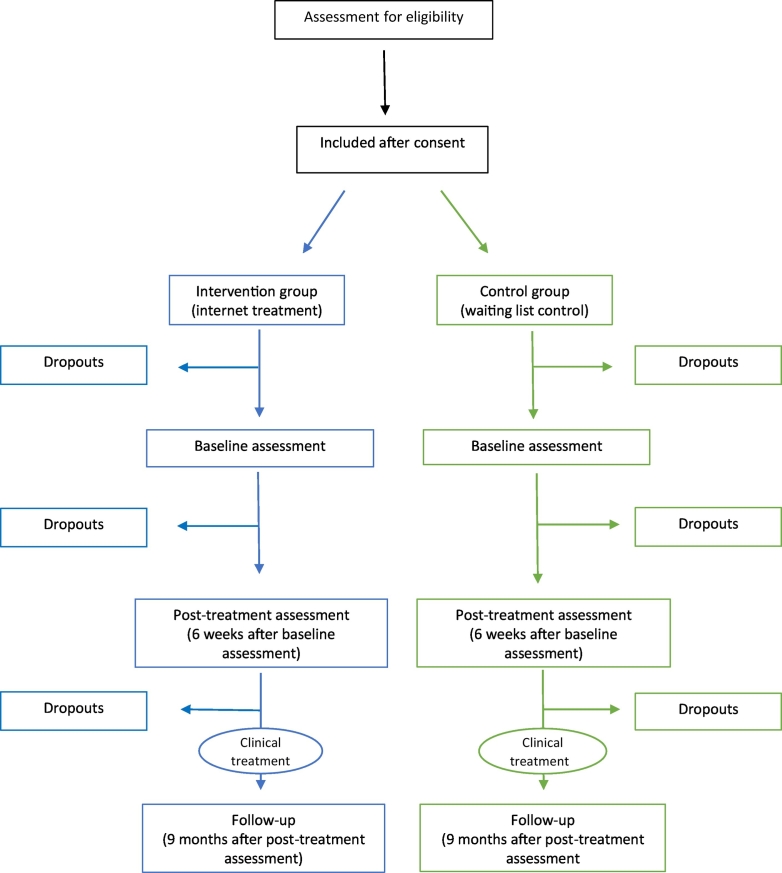
Flowchart of the study.

Online assessments are conducted at baseline (T0), posttreatment (6 weeks after randomisation, T1), and at follow-up (9 months after randomisation, T2). Self-reported online questionnaires generally have the same psychometric properties as paper-and-pencil administrated questionnaires (Buchanan, 2003). Three emails are sent as reminders for each assessment. A flowchart of the study is presented in Fig. 3. Among the first 15 participants, the drop-out rate between baseline and post-treatment is 33%, while 47% of those participating in post-treatment are lost to follow-up.

Medical records will be assessed after proper authorization by the clinic, in accordance to the ethical application, to control for parallel clinical treatment during the intervention period.

### 2.7 Sample size calculation

Sample size calculation was based previous clinical experience based on self-reported vulvar pain using a Visual analogue scale, which is a Likert scale between 0 and 10, where 0 represents no pain and 10 represents the worst possible pain; this is regularly assessed with questions on pain at intercourse or tampon insertion, which are posed during the first appointment with gynaecologists at Uppsala University Hospital. A minimum of 26 patients in each group at each time point is estimated to give 80% power for ascertain a difference between the groups at a significance level of *p* < 0.05 for a minimum clinically significant improvement of at least 1.2 units in the Visual analogue scale for vulvar pain, as described above, with an estimated 20% drop-out rate.

### 2.8 Study outcomes

#### 2.8.1 Primary outcomes

Pain intensity at vaginal intercourse and tampon test is assessed using an 11-point Likert numeric rating scale (Foster et al., 2009; Wammen Rathenborg et al., 2019). Self-reported pain is rated between 0 (”no pain”) and 10 (”worst possible pain”). The tampon test has a fair to moderate week-to-week test-retest reliability and is significantly associated with pain at intercourse and pain intensity (Foster et al., 2009).

Impact of pain on sexual function is assessed by asking “How do you rate your pain today?” and is rated from 0 to 3, where 0 corresponds to never having pain at vaginal intercourse and 3 corresponds to severe pain that often/always prevents intercourse.

#### 2.8.2 Secondary outcomes

Secondary outcomes include pain acceptance, depression, anxiety, sexual function, and quality of life. To better our primary outcome measures, two other variables will also be analysed: unwillingness to perform the tampon test and abstinence from vaginal intercourse, both measured as binary variables.

Chronic pain acceptance questionnaire (CPAQ) is a self-assessment form used for patients with chronic pain and measures pain acceptance (Feliu-Soler et al., 2018; McCracken et al., 2004; Reneman et al., 2010). CPAQ is divided into two subscales: activity engagement (participation in activities despite living with pain) and pain willingness (attempts to avoid and control pain as pain management (ineffective coping) (Feliu-Soler et al., 2018; McCracken et al., 2004; Wicksell et al., 2009). The form consists of 20 statements that can be rated between “never true” to “always true”. CPAQ has good internal consistency and similar psychometric properties both when administered online or via paper-and-pencil (Fish et al., 2010; Wicksell et al., 2009).

The Female Sexual Function Index (FSFI) is an instrument for measuring female sexual function and consists of 19 questions within 6 domains: sexual desire, arousal, lubrication, orgasm, satisfaction, and pain (Rosen et al., 2000; Wiegel et al., 2005). Questions are rated on a Likert scale from 0 or 1 to 5, where 0 corresponds to no attempt at intercourse. Higher FSFI scores indicate better sexual functioning (Ter Kuile et al., 2006). Test-retest reliability has appropriate level of stability, while the internal consistency of the instrument and its subscales are satisfactory to good (Ter Kuile et al., 2006). The ability of the scale to predict the presence or absence of sexual complaints is considered excellent (Ter Kuile et al., 2006).

The Female Sexual Distress Scale-Revised (FSDS-R) is an instrument for measuring female sexual distress and consists of 13 questions (Derogatis et al., 2008). The score varies between 0 and 48 and each question can be rated from 0 (never) to 4 (always) (Derogatis et al., 2008). Higher FSDS-R scores indicate more sexual distress (Ter Kuile et al., 2006). The instrument has excellent reliability and good discriminant validity (Derogatis et al., 2008; Ghassami et al., 2014).

The Beck Anxiety Inventory (BAI) is a scale that measures anxiety using a list of 21 anxiety symptoms and has high reliability and good validity (Beck et al., 1988; Muntingh et al., 2011). The score varies between 0 and 63 and symptoms are rated from 0 (“not at all”) to 3 (“severely, I could barely stand it”) (Muntingh et al., 2011).

The Montgomery-Åsberg Depression Rating Scale (MADRS-S) is a 9-item scale for measuring depressive symptoms and has satisfactory internal consistency (Fantino and Moore, 2009). The score varies from 0 to 54 and symptoms are rated between 0 and 6 (Hollandare et al., 2010). Higher scores indicate more depressive symptoms. A high correlation has been found between administration of the survey via the internet and using paper and pencil assessments (Hollandare et al., 2010).

The Revised Dyadic Adjustment Scale (rDAS) is an instrument for measuring dyadic adjustment in relationships. It has acceptable levels of construct validity and adequate internal consistency (Busby et al., 1995; Crane et al., 2000). The score ranges from 0 to 69, where a score of 48 is the cut-off point between distressed and non-distressed couples (Crane et al., 2000).

The Satisfaction with Life Scale (SWLS) is a 5-item scale for measuring life satisfaction. Each item is scored from 1 to 7 and the possible range of total scores varies from 5 (low satisfaction) to 35 (high satisfaction). A score of 20 represents a neutral point in the scale and higher scores indicate greater satisfaction with life (Diener et al., 1985; Pavot and Diener, 2008). The scale has good reliability and acceptable validity (Hultell and Petter Gustavsson, 2008; Maroufizadeh et al., 2016).

The EQ-5D (EuroQol-5-dimension questionnaire) is an instrument for measuring quality of life and consists of two parts: one part with 5 questions about different dimensions of health-related quality of life, and one part with a Visual Analogue Scale where the respondent assess their overall health status (Aubry et al., 2017; Janssen et al., 2013). The instrument has good construct validity and excellent responsiveness to change (Aubry et al., 2017).

Other collected data are sociodemographic information, exposure to violence, and social support. The ENRICHD Social Support Instrument (ESSI) is a 7-item self-reported instrument for measuring perceived social support (Vaglio et al., 2004). A 5-item Likert scale ranging from “none of the time” to “all the time” is used (Lett et al., 2009). Higher scores indicate high social support (Vaglio Jr et al., 2004). The instrument has high reliability and acceptable validity (Vaglio Jr et al., 2004). The number of visits related to vulvodynia will be retrieved from participant's medical records.

All measures will be collected at T0, T1, and T2, except rDAS (collected only at T0 and T2). Sociodemographic information and answers to questions about exposure to violence, general pain, and comorbidities are collected at T0.

Patients receive a small value gift certificate when they have filled out T1 and another one when they have filled out T2.

### 2.9 Statistical analysis plan

Linear mixed effect models will be used to assess the effect of the internet-based treatment on vulvar pain, pain acceptance, depression, anxiety, sexual function, and quality of life over time, by applying the intention-to-treat approach. Variables that vary significantly between groups at baseline will further be adjusted for in the model. Linear mixed modelling is a frequently recommended method for its abilities to handle missing data and correlated observations in repeated-measures data (Gueorguieva and Krystal, 2004; Yoo, 2010). The best-fitted model to the data consists of an unstructured covariance matrix using the restricted maximum likelihood (REML) estimation procedure. Baseline, posttreatment and follow-up measures will be used as repeated measures and intervention/control group will be used as fixed factors.

As significant drop-out is common in internet-based interventions, further analyses will be performed. Continuous data will be analysed with general linear models using besides intention-to-treat also per protocol approaches to assess the effects of the intervention on vulvar pain, depression, anxiety, sexual function, and quality of life at different time points (post-treatment and follow-up). Last observation carried forward (LOCF) will be used for the intention-to-treat analysis. Complementary analysis using worst case imputation and analysis using a per-protocol approach without imputation will also be carried out. Effect size will be calculated with Cohen's d. *P* values less than 0.05 will be considered statistically significant. Ordinal (impact of pain on sexual function) and binary data (willingness to perform tampon test and attempt at vaginal intercourse) will be analysed with Mann Whitney's test, Fischer's exact test and multivariate logistic regression, respectively, to assess differences between groups at posttreatment and follow-up. The binary data are referring to secondary outcomes and will be analysed using a per protocol approach only. Descriptive statistics at baseline and the corresponding changes during follow-up both will be provided in tables and graphically. All analyses will be completed using the IBM SPSS statistical package.

## 3 Patient and public involvement

This study is performed without participant involvement apart from the feedback received during the pilot phase (data not published), that was implemented in order to assess the feasibility of the study. The pilot study was conducted among patients who had contacted the hospital in the past because of vulvodynia. The study content was adjusted after comments from these participants. Otherwise, participants in the present study are not invited to comment on the study design, interpret the results, or contribute to the writing or editing of this document for accuracy.

## 4 Ethics, data management and dissemination

The EMBLA study has been approved by the regional ethical review board in Uppsala, Sweden (registration number 2015/031), and is registered at clinicaltrials.gov (protocol ID EMBLA, ID NCT02809612). The study is conducted following the principles of the Declaration of Helsinki.

In addition to the information provided over the telephone, written information about the EMBLA study is sent to all women who are interested in participating in the study. Eligible participants are asked to provide written informed consent. Each participant is assigned a code and the data included in the database cannot be traced back to any individual participant. All data are thus coded and stored securely on Uppsala University's servers. The code key is stored separately on a different folder in the secure server. All consent forms are archived in locked spaces.

The results of this trial will be disseminated in peer-reviewed publications and as presentations at international conferences.

## 5 Discussion

Internet-based treatment would also enable participants from different and often remote geographic areas to receive treatment. In Sweden, most of 97% of the population has access to fast internet (Internet World Stats, 2019). The study's results are expected to be of value in the planning of clinical care in the medical area.

As a randomised controlled trial with short- and long-term follow-up points, the EMBLA study intends to provide a novel and better understanding regarding the treatment of vulvodynia and the role of internet-based treatment as a complement to standard care for women suffering from vulvodynia. The use of a randomised controlled design and the use of well-accepted validated self-assessment questionnaires are strengths in our study.

Selection from a clinical setting and access to participants' medical records to control for concomitant treatments and other variables is also a strength in our study. The risk for diagnostic misclassification is low, as the diagnosis of vulvodynia has to be confirmed in a screening interview. Guidance may facilitate adherence to treatment and is important to increase efficacy (Baumeister et al., 2014; Webb et al., 2017). A limitation may be the eCoaches varying professional experience with CBT treatment with ACT components. However, the qualification of eCoaches has minor importance for the outcome (Baumeister et al., 2014). Further, the differences in previous experiences are minimised by using message templates and by the possibility to discuss with senior researchers (Lattie and Graham, 2019).

Difficulties in recruiting are common in clinical trials and many trials may take longer to complete recruitment than primarily planned (Sully et al., 2013). Nevertheless, loss to follow-up seems to be a problem among internet interventions trials (Mathieu et al., 2013) and high dropout rates and low retention rates are a known issue among iCBT trials (Webb et al., 2017). An additional limitation of our study is the time period needed to conclude the recruitment phase, which has been prolonged due to dropouts and difficulties with retaining participants. To manage the issue of missing data, a per protocol approach will be used as a complement to ITT to facilitate interpretation of the findings (Beckett et al., 2016). Whilst ITT analysis has the advantage to preserve the balance afforded by randomisation, minimising the risk of for type I error and resembling clinical practice (Beckett et al., 2016; McCoy, 2017), a per-protocol approach may provide the true efficacy of an intervention among completers. LOCF is a common imputation method, but it has previously been criticized for overestimation of values as the true mean differences are not present when dropout rates differ between groups (Gueorguieva and Krystal, 2004; Lachin, 2016). Therefore, worst case imputation will be used to avoid inaccurate estimates and ensure the trustworthiness of the results.

Further, the specific limitations and practical problems encountered in this study may be of use for clinicians planning to implement this material. These should be taken into consideration when adjusting the content for use in clinical settings. A study involving qualitative analysis would be of value to further explore this issue and is now under way, which may have an impact on the planning of future studies.

At this point, there is a considerable knowledge gap when it comes to internet-based treatment for patients with vulvodynia. If effective, internet-delivered psychological treatment can easily be adapted and used in clinical settings to complement clinical treatment in a primary care setting.

## CRediT authorship contribution statement

AS designed the study together with UH, MB, SH, and IJ. Data acquisition, analysis, and interpretation were carried out by AHE, AS, UH, and MK. All authors revised and approved the final version of the article.

## Declaration of competing interest

The authors declare that they have no known competing financial interests or personal relationships that could have appeared to influence the work reported in this paper.
